# PATTERNS OF MARIJUANA USE IN THE US OLDER ADULT POPULATION

**DOI:** 10.1093/geroni/igad104.0564

**Published:** 2023-12-21

**Authors:** Jennifer Ailshire

**Affiliations:** University of Southern California, Los Angeles, California, United States

## Abstract

Marijuana use is increasing in the U.S. population, including among adults 50 and older, but research on population characteristics of marijuana use in middle aged and older adults is limited. We use data from the 2018-2021 Behavioral Risk Factor Surveillance System (BRFSS) surveys, which are representative of U.S. state populations, to estimate prevalence and frequency of marijuana use by age. The data are from 39 states that included the module on marijuana use in the past 30 days (n=466,469). We also examine age patterns in use by legalization status and reasons for use of marijuana. Reported marijuana use is much lower among those ages 50 and older (6.49%) than 18-49 (16.3%). Multivariable regression analyses show that marijuana use declines with age (p<.001) and is lower among women (p<.001). Marijuana use among older adults is slightly lower in states where it is illegal compared to states that have legalized (4.6% vs. 7.1%, p<.001). Use of marijuana for medical reasons increases with age; nearly two-thirds of adults 80+ who use marijuana report doing so for medical reasons. Adults 80+ also report the highest frequency of use, likely reflecting greater medical need. The most common conditions reported among adults 50+ who use marijuana for medical reasons are arthritis (61%), depression (36%), asthma (20%), and COPD (18%). Our prevalence estimates of use at older ages match those from other national surveys, but our findings suggest older adults are using marijuana largely for medical reasons.

